# Fairness, Trust, and Well-Being Among Young Adults: Evidence from 2021 Chinese General Social Survey (CGSS)

**DOI:** 10.3390/healthcare12212186

**Published:** 2024-11-03

**Authors:** Liyun Wu, Gang Wang

**Affiliations:** 1 The Ethelyn R. Strong School of Social Work, Norfolk State University, Norfolk, VA 23504, USA; lwu@nsu.edu; 2 School of Humanities and Communication, Ningbo University, Ningbo 315020, China

**Keywords:** Chinese general social survey, fairness, factor analysis, trust, well-being

## Abstract

**Background**. As China has undergone tremendous socioeconomic and demographic changes during and after the pandemic, it is crucial to enhance youth well-being and facilitate their transition into the workforce. This study aims to explore the multi-dimensional features of well-being and examine their relationships with fairness and trust. **Methods**. Using the nationally representative data from the 2021 *Chinese General Social Survey* (CGSS), this study analyzes the well-being of 1726 young adults aged 18 to 34 years old living in 19 provinces in China. **Results**. The research findings generate five latent factors of well-being and reveal the multi-faceted nature of subjective well-being, including physical and mental health challenges, positive outlook, sense of purpose, personal growth, and self-fulfillment. Physical and mental health is the cornerstone for well-being and happiness. Young people with poor physical and mental health conditions are less likely to perceive that society is fair or that people are trustworthy. Additionally, a positive outlook and future orientation are strongly associated with high fairness and/or trust. **Discussion and Conclusions**. By investigating the differential domains of well-being, this study contributes to the literature with the new conceptualization of well-being sub-measures and their application to youth. Furthermore, this study identifies that well-being is not only an individual-level characteristic but also a group-level attribute that can contribute to fairness and trust at the societal level. As China continues to expand its infrastructure on education, health, and technology, it is expected that sustainable economic growth and development should boost young people’s career development and promote their upward mobility.

## 1. Introduction

Well-being has increasingly become the goal of public health and public policy locally, nationally, and globally. The World Health Organization (WHO) defines well-being as “a positive state experience by individuals and society. Well-being encompasses quality of life and the ability of people and societies to contribute to the world with a sense of meaning and purpose [1]”. The *World Happiness Report* [2] suggested that the success of countries should include the happiness of people and that happiness needs to become an operational objective for governments. In 2022, the WHO published a global framework to integrate well-being into public health and emphasized the importance of promoting health and well-being via health promotion programs such as health literacy and community engagement [3]. By developing a holistic approach to incorporate both material and non-material values, well-being studies can assess the social, ecological, and economic progress of society.

The measure of well-being often overlapped with mental health indicators. The World Health Organization Five Well-being Index (WHO-5) is widely used to measure subjective well-being, which includes five items (e.g., being happy, being interested in things, having lots of energy, being calm and peaceful, and being fresh and rested) with an emphasis on positive effects. These five questions commonly overlap with other mental health instruments, such as the *Medical Outcome Studies* (MOS) *Short-Form* 36 (SF-36) *Health Survey* and the mental health component of the *International Classification of Disease 10th revision* (ICD-10). Well-being, happiness, or life satisfaction have both practical and theoretical significance on the individual at institutional, local, national, and global levels. The profile of well-being is pivotal in decision-making and policy changes and has implications for health and wealth across study populations in society.

It is critical to foster youth mental health and well-being in China as China has undergone drastic changes in demographic structure and economic transformation during and after the COVID pandemic. With increasing stress and competition in school and job markets, young people during the pandemic experienced isolation, loneliness, stress, and disruption in relationships [4]. According to a report from the Asian Development Bank, one out of every five young people in China in 2022 during COVID was unemployed [5], which sets a record-breaking high for Chinese unemployment, and this challenging situation has drastically changed young people’s outlook on the future. Another source of data from the World Bank revealed the unemployment rate among youth: the percent of labor at ages 15–24 has risen from 10.7% in 2019 prior to COVID to 15.7% in 2023 post-COVID [6]. Despite the variation in the age ranges of youth and rates of unemployment from different studies, youth unemployment was undoubtedly higher than what it used to be prior to COVID. The conjecture of job scarcity, widespread layoffs in white-collar sectors, and an unprecedentedly high number of college graduates all contribute to the psychological well-being of Chinese youth.

This study has two research aims. First and foremost, this project aims to fill the gap in earlier research by conducting an exploratory factor analysis of a unique 21-item well-being scale based on a sample of young adults from the 2021 *Chinese General Social Survey* (CGSS). Additionally, the research aims to examine the relationship between various domains of well-being, fairness, and trust. By investigating the differential domains of well-being, this study contributes to the well-being literature with a new conceptualization of well-being sub-measures and their application to young people.

## 2. Literature Review

### 2.1. Well-Being, Happiness, and Life Satisfaction

Happiness, well-being, and life satisfaction are indicators of the quality of human life. The well-being literature is abundant in terms of identifying relationships between well-being and an array of correlates. The first line of research considers data on well-being, life satisfaction, or happiness as outcome measurements when assessing the effect of policy changes on well-being. Examples included cigarette taxes on welfare [7] or the effect of socioeconomic status on well-being [8,9,10,11,12]. When predicting well-being, income, or monetary resources, this stands out as one of the most influential factors. There are many answers to questions like “Does money buy happiness?” Although the debate on the association between income and happiness is endless, many existing empirical studies have recognized a positive association between income and happiness. Increases in income are desirable from an individual’s perspective. Using 10-year longitudinal data among 4942 American adults, Diener et al. [13] analyzed the effect of absolute income on subjective well-being. They found that absolute income, which was spent on necessary needs, increased subjective well-being, and yet the rate of increase was smaller at higher income levels. Gardner and Oswald [14] demonstrated that greater material riches created greater mental well-being among adults living in England. Their results identified that a random sample of Britons who received medium-sized lottery wins revealed better psychological health in comparison to those with no wins or small wins. Additional studies further identified the effect of income on well-being varied by age, and results from a national sample of 2018 China Family Panel Studies (CFPS) reported the wealthy old Chinese as the happiest group [15].

Not only is the positive association between income and well-being found in individual participant data, but a similar relationship is also identified in empirical studies using country-level data by evaluating economic growth and the average subjective well-being of members of society. In 2008, Stevenson and Wolfers [16] analyzed this relationship by evaluating *World Value Survey* country-wave data from 1994–2004 for Japan and Europe and the *Current Population Survey* from 1972–2005 for the United States. Their results suggested that wealthier nations had greater subjective well-being than less affluent nations, and better-off citizens of a nation were happier than their less well-off counterparts.

In addition to the direct association between money and well-being, studies also investigated the importance of “comparison income”, the relevance of the income of a reference group, on individuals’ subjective well-being. Using the subsample of 1992–1997 German Socio-Economic Panel (GSOEP) data, Ferrer-i-Carbonell (2005) found that individuals’ happiness was based not only on their own income but also the income of the reference group to whom they compare. Adults were happier if their income was higher than that of the reference group.

Furthermore, researchers have differentiated the broad concept of well-being into two forms: “experienced well-being” versus “evaluative well-being”. Experienced well-being refers to people’s feelings about their daily activities, experiences, engagements, and moments, while evaluative well-being is related to a retrospective evaluation of their lives and overall life satisfaction by Killingsworth in 2021 [17]. Although it is not surprising that many results resonate with the positive relationship between income and well-being regardless of the type of well-being, some results discovered the subtle differences between evaluative and experienced well-being. Well-being rose with income at all levels, which was verified with multiple datasets from both rich and poor nations and affluent and less-affluent citizens within the country [16], and high income raised life satisfaction [15]. However, income does not necessarily improve experienced well-being or emotional well-being. By analyzing responses from more than 450,000 American residents in 2008–2009, Kahneman and Deaton identified that people who earned less than USD 75,000 enjoyed more happiness as income grew or they were emotionally affected by misfortunes such as poor health, loneliness, marriage breakdown, and this positive relationship disappeared when they earned more than USD 75,000 [18]. Jebb and colleagues identified another income cutoff to differentiate the impact on evaluative well-being versus emotional well-being. After analyzing the data from the Gallup World Poll with a sample of more than 1.7 million participants across the globe, they identified that the income cutoff existed at USD 95,000 in terms of evaluative well-being and between USD 60,000 and USD 75,000 for emotional well-being [19].

In addition to wealth and income, other factors such as physical health and mental health are also essential for well-being. Chen and colleagues analyzed a sample of 2598 US adults who responded to a 12-item well-being measure with six domains, including happiness, health, meaning, character, relationships, and financial stability [20]. Each domain consists of two indicators, which are self-reported on a scale of 0 to 10. Their findings suggest that US younger adults had lower self-rated physical health and a higher incidence of loneliness compared to other age groups. Social disconnection is a threat to well-being [21]. The fact that health is a contributing factor to well-being and happiness is also observed in other non-US samples, such as immigrants to Europe [22].

### 2.2. Trust and Fairness

Trust has been studied countlessly over time across disciplines, including health and medicine [23], business [24], liberal arts [25,26,27,28], and science and engineering [29,30,31], just to name a few. Despite there being no universally accepted definition, trust is commonly perceived as a multifaceted and layered construct that is essential for building a fair and equitable society. For instance, trust is commonly viewed as relationships between individuals or between individuals and groups. At the micro level, trust between people can ensure people thrive and are safe from harm [32]. At the macro level, trust-based cooperation is key to facilitating social interactions and the foundation for thriving families, groups, organizations, and institutions, both locally and globally. Trust is essential to stimulate cooperative economic behavior and promote nation-wide economic development [33]. Trust is vital for business success as it is intertwined with all aspects of business operations, ranging from human interactions and cross-cultural communications to large capital investment and high technology innovation [34]. Trust is also fundamental for civil liberties, transparency, political representation, and the eradication of corruption [35]. Trust and trustworthiness lay the foundation for knowledge generation and research innovation [36]. The level of trust is a determinant for strong family functioning as it relates to family members’ perceptions and assumptions of how other family members will act and respond [26,37].

Scholars have presented empirical evidence uncovering influential factors associated with trust. Using the *Canadian General Social Survey* (GSS), Helliwell and Wang examined several measures of trust, including general interpersonal social trust, trust in co-workers, trust in neighbors, trust in strangers, and the likelihood of a lost wallet containing CAD 200 to be returned if found [38]. Their research findings suggest that relationships play a vital role in trust. Factors such as the number of close friends, the number of close relatives, the frequency of seeing close friends, and the frequency of seeing close relatives are positively associated with trust. Relationships are also positively associated with well-being, which was measured using a self-rated life satisfaction question on a scale of 1 to 10.

In a similar way, there are many definitions of fairness across disciplines. The debate of “fair or just or right” is endless in human history. In addition, the variation in fairness is substantial across cultural norms, geographic boundaries, economic resources, technological access, legal and political systems, as well as other social and historical contexts. In the pursuit of a fair society, people need equal access to opportunities, everyone in society is given the chance to move upward, the needs of the most disadvantaged population are met [39], and there are procedural justices to manage disputes and protect fairness [40].

The literature supports a link between fairness/justice and subjective well-being. Cavazos utilized the *World Values Survey* to examine the relationship between autonomy support and life satisfaction for a sample from three countries: Argentina, Brazil, and Mexico [41]. The findings suggest that life satisfaction is positively associated with autonomy support. In a more just society, there are fewer obstacles for members of society and members can exhibit more autonomy to have their needs met. This positive correlation exists at the macro level too. After analyzing panel data from European countries, Kavuri and Shao (2017) identify that national-level social justice positively influences economic performance [42].

### 2.3. COVID-19 Lockdown in China

The World Health Organization declared the outbreak of COVID-19, an infectious disease caused by the severe acute respiratory syndrome coronavirus 2 (SARS-CoV-2), a pandemic on March 11, 2020 (WHO, n.d.). As the virus spread rapidly from person to person, China initiated a zero-COVID policy by implementing lockdown restrictions nationwide. The COVID quarantine policies and procedures generated long-lasting impacts on young people at various stages of their lives. For college students who lived in dormitories with 4–6 students per room, they had to adjust to new norms such as prohibition of indoor gatherings, closure of campus facilities (e.g., libraries, restaurants, gyms, clubs, classrooms), adoption of online teaching and learning, interruption of social activities, and travel bans preventing them from friend or family visits or job seeking. All these stringent practices significantly influenced their physical health, such as restless sleep, poor dietary habits, appetite changes, and missed doctor’s visits, as well as affected their mental health as a result of isolation, loneliness, stress, and disruption in relationships [4]. Unsurprisingly, these challenges encountered by Chinese college students were also observed among students in other areas of the world. Physical and social distancing practices caused depression, loneliness, stress, reduced motivation, job loss worries, sleep disorder, and appetite changes among US students [43]. A reduction in overall physical (worse sleep quality) and mental health (isolation and depression) was also identified among students in Europe [44,45].

### 2.4. Conceptual Framework

This study applies the psychological theory of motivation and integrates it in analyzing the mechanism between happiness and fairness/trust. Maslow’s renowned theory of “hierarchy of needs” conceptualizes a hierarchical pyramid of human needs ranging from physiological and safety to psychological needs and emphasizes the importance of well-being. The current study of Chinese young adults will add empirical evidence to the robustness of Maslow’s conceptual framework.

What makes people happy? Happiness cannot be obtained without having needs met. According to Maslow’s theory [46], human needs can be categorized into five levels in a pyramid shape. The foundation level is related to physiological needs such as food, drink, and sleep. The next level is safety needs, including not only physical safety but also economic, social, and psychological security, which empower individuals with resources to survive difficult times. The third high level is love and belonging. Good human relationships such as family and friends and active engagement with surrounding communities make people feel loved, and this sense of belonging is critical to sustaining them through challenges. The next high level is self-esteem. At this level, individuals are eager to be recognized for their unique talents and capabilities, which boost their confidence and cause others to respect them. The highest level of human needs is self-actualization. At this top stage, self-actualizing people have the realization of their full potential, full development of their abilities and appreciation for life, creative expression, and a grounded sense of well-being and satisfaction with life. Although Maslow described a full range of individuals’ psychological development in a hierarchal way, it does not mean that the pursuit of happiness has always followed this gradual change with small steps in a straight-line mode. An integrated relationship between each of these five stages is inevitable, and individuals must constantly adjust to their real-life situations.

## 3. Methods

### 3.1. Data and Sample Participants

The *Chinese General Social Survey* (CGSS) is the national representative survey administered by the leading Chinese academic institutions, which has surveyed Chinese citizens for more than two decades. The CGSS survey aims to collect quantitative data to measure the growing complexity of society and provide a national source of information for policymakers, researchers, educators, and practitioners from all walks of life. Comparable to the *General Social Survey* (GSS), which was launched by the University of Chicago in 1972, the CGSS data started in 2003 and are administered by Renmin University of China and funded by the University’s 985 science research programs. Fourteen waves of data have been collected and released for public use, including four waves from 2003 to 2008, nine waves from 2010 to 2019, and one most recent wave in 2021. Data were collected for a wide range of topics, including childbearing, education, employment, health, income, family structure, elderly care, neighborhood and community, political participation, quality of life, social structure, attitudes, social trust, social issues, technology penetration, climate change, environmental problems, and other important social issues. Using a multistage stratified probability sampling method, this survey can reach out to both urban neighborhoods and rural villages to reflect the diverse population and great physical and geographic complexity of the country.

This study utilized a subsample of the cross-sectional data from the 2021 CGSS, which includes 1726 participants ages between 18 and 34 years old representing 19 provinces, autonomous regions, or municipalities in China (referred to as “provinces” hereafter). The inclusion criteria of the sample selection has two aspects: (1) an age between 18 and 34, and (2) a subjective happiness score in the range of 1 to 10. This study is based on secondary data, which are easily accessible for public use.

### 3.2. Measures

*Happiness and Well-Being Instrument*. This happiness and well-being instrument includes twenty-one items on a 6-point Likert scale with 1 = strongly disagree, 2 = disagree, 3 = somewhat disagree, 4 = somewhat agree, 5 = agree, and 6 = strongly agree. Participants were asked to evaluate their level of agreement for each of these items. They are (1) society provides more opportunities, (2) I become more capable over the years, (3) my life goals are fulfilling, (4) I am mostly idle, (5) I am unclear of how meaningful what I have done in my life is, (6) I fear body functions, (7) I am content compared to my peers, (8) I am satisfied with my family’s income, (9) I am worried about small things, (10) I am upset with my physical health, (11) I have a hard time maintaining friendships with others, (12) I like my personality, (13) I have fewer friends than most people, (14) I am happy staying with my family, (15) I have worse luck than most people, (16) I am confident with societal development, (17) I am at a disadvantage compared to others, (18) I lose spirit when encountering unhappy things, (19) I have become more mature over time, (20), I have difficult times with my family, (21) I am satisfied with the natural environment.

*Fairness and Trust.* To measure fairness, sample participants were asked to evaluate the fairness of the society on a 5-point Likert scale: 1 = completely unfair, 2 = moderately unfair, 3 = neutral, 4 = moderately fair, and 5 = completely fair. To measure trust, respondents were asked to evaluate whether most people in society can be trusted on a 5-point Likert scale: 1 = strongly disagree, 2 = moderately disagree, 3 = neutral, 4 = moderately agree, and 5 = strongly agree.

*Demographic and Socio-Economic Variables.* In this study, fairness and trust are outcome variables. Fairness is categorized into 3 levels: unfair, neutral, and fair. Trust is also categorized into 3 levels: disagree, neutral, and agree. Demographic variables include gender (0/1), age in years old, and ethnicity as binary, with 1 indicating majority Han Chinese and 0 representing ethnic minority people (Zhuang Chinese, Hui Chinese, Korean Chinese, Miao Chinese, Manchu Chinese, Mongol Chinese, Tibetan Chinese, or others). Socioeconomic variables include educational attainment, self-reported health, and marital status. Education has 4 categories, including middle school or less, high school or equivalent, associate degree, and college. Self-reported health is categorized into three levels: poor or fair, moderately healthy, and very healthy. Marital status is dichotomous with never married versus other statuses. Due to too many missing values, income and employment status indicators are not included in this study.

### 3.3. Analytical Strategies

This study utilizes multivariate analytical strategies to evaluate the relationship between variables. Factor analysis, a data reduction tool, is utilized to identify the underlying latent constructs or dimensions of the happiness and well-being instrument. Exploratory factor analysis reduces a number of observable variables into a few unobservable dimensions. By using the orthogonal rotation method (varimax), each dimension is independent of another, whereas all the variables within each dimension are correlated with each other. Multiple regression is used to discover the relationships between fairness and well-being and trust and well-being, respectively. All the analyses are performed using the latest version of statistical software SPSS 29.0.

## 4. Results

### 4.1. Descriptive Statistics

This study includes 1762 young adults, and their average age is 26.39 years old (SD = 5.092), with the youngest being 18 and the oldest being 34 years old. As shown in Table 1, there are more females (54.9%) than males (45.1%). Han Chinese people comprise 91.7% of the sample, and ethnic minority Chinese people account for 8.3%. In terms of educational attainment, 44.8% of the sample have only secondary education (21.2% middle school or less, and 23.6% high school or equivalent, respectively), 16.6% have associate degrees, and 38.6% have college and above degrees. Self-reported health is distributed as poor or fair (20.1%), moderately healthy (45.3%), and very healthy (34.6%). More than half of the respondents have never been married (55.6%), and the remaining have another marital status. Table 2 presents the descriptive statistics of the happiness and well-being instrument. Scores of each item range from 1 to 6, with the lowest mean value of 1.65 (SD = 0.903) and the highest mean value of 4.86 (SD = 0.730).

### 4.2. Results from Factor Analysis

Exploratory factor analysis (EFA) generates and retains five factors that have eigenvalues greater than one. These five factors are named as follows: physical and mental challenges (factor 1), positive outlook (factor 2), sense of purpose (factor 3), personal growth (factor 4), and self-fulfillment (factor 5). These five factors capture five dimensions of happiness and well-being.

Sample adequacy is measured by the Kaiser–Meyer–Olkin statistic with a value of 0.855 and Bartlett’s test of sphericity with the approximate Chi-square value of 3156.330 (*p* < 0.001). Variables are correlated, and the sample is adequately ready for factor analysis.

Table 3 presents the rotated factor loadings for happiness. These factor loadings indicate the correlations between the variable and the factor. All the low factor loadings (<0.4) are removed from the table, which makes the output easier to read. The remaining clusters of high correlations clearly indicate the strong association between each factor and correlated variables.

For the physical and mental health challenges dimension, the largest factor loading is 0.793 and the smallest factor loading is 0.481. For the positive outlook dimension, factor loadings range from 0.543 to 0.797. For the sense of purpose dimension, factor loadings range from 0.458 to 0.811. For the personal growth dimension, factor loadings range from 0.498 to 0.680. Lastly, for the self-fulfillment dimension, factor loadings are 0.612 and 0.812.

Furthermore, these five factors cumulatively account for more than half of the variance (53.44%) in the happiness and well-being instrument. As shown in Table 4, the first factor accounts for 25.08% of the total variance of the data, the second factor is 12.23% of the total variance, the third factor 6.14% of the total variance, the fourth factor 5.12% of the total variance, and the fifth factor 4.87% of the total variance.

### 4.3. Results from Multiple Regression

The results of the two multiple regression models are shown in Table 5 and Table 6. When controlling for socio-economic characteristics, physical and mental health challenges are negatively associated with fairness (*p* = 0.007), implying that young adults who have physical and mental challenges are less likely to value fairness. However, young adults who have a positive outlook (*p* < 0.001) and self-fulfillment (*p* = 0.004) are more likely to perceive that society is fair. This model has an R-square value of 0.071, implying that five new factors and background characteristics altogether account for 7.1% of the variance in the fairness measure.

Similarly, after controlling background characteristics, physical and mental health challenges are also observed to be negatively associated with trust (*p* = 0.002). In addition, a lack of sense of purpose was negatively associated with trust (*p* = 0.014). Lastly, it was noted that the positive outlook dimension contributed to the formation of trust (*p* = 0.006). Young adults who are college educated are more likely to trust people (*p* = 0.040) compared with people with lower levels of educational attainment. The second regression model has an R-square value of 0.063, implying that five new factors and background characteristics account for 6.3% of the total variance in the trust variable.

## 5. Discussion

This research project analyzed a set of subjective well-being questions for a nationally representative sample of 1726 young adults aged 18–34 years old using the 2021 *Chinese General Social Survey* (CGSS) data. This study identifies that subjective well-being is a multi-faceted and layered construct with five dimensions: physical and mental health challenges, positive outlook, sense of purpose, personal growth, and self-fulfillment. Physical and mental health disparities shaped young people’s perception of fairness and trust within society. Those with poor physical and mental health were less likely to agree that society is fair or that people are trustworthy. However, the findings report college education is a vital source of social trust, and education generates higher trust in others. Young people with a positive outlook were more likely to trust people and perceive society as fair. A positive outlook can enhance their resilience and capabilities to handle uncertainties, challenges, and critical decision-making moments.

Empirical evidence from the current study aligns with the existing literature. Young people in China during the COVID period experienced mental health challenges [46,47], and their well-being was associated with social trust [48]. However, those existing studies are only based on data collected from one province, and none of them are nationally representative. This paper thus fills in the gaps of previous studies and adds to the existing body of knowledge by analyzing a nationally representative sample. In addition, the empirical evidence from this study fits Maslow’s Hierarchy of Needs theory well [49]. The results from the factor analysis of the happiness instrument suggested five latent constructs: physical and mental challenges, positive outlook, sense of purpose, personal growth, and self-fulfillment. The first latent factor (i.e., physical and mental challenges) is reflective of three basic tiers in Maslow’s Hierarchy of Needs theory. Chinese young adults with an average age of 26 years old clearly indicated that their biological, physical, and behavioral health conditions were the foundation of their happiness and well-being. In addition, happiness is grounded in maintaining healthy relationships with friends and people in the community. Lack of social connections and stable relationships decreases the level of happiness and the sense of security, and the need for love and belonging cannot be met. Chinese young adults also linked their happiness with higher tiers of psychological development: the need for self-esteem and self-actualization. Four latent factors (i.e., positive outlook, a sense of purpose, personal growth, and self-fulfillment) depicted various aspects of self-esteem and self-actualization. Chinese young adults perceived their financial security as one of the most important indicators of self-actualization.

Lack of employment opportunities and income-generating activities during the COVID pandemic, along with the slowing economic growth due to structural factors such as a shrinking labor force, an aging population, slowing productivity, a decline in nongovernment investment growth, and a severe contraction in the housing market [50,51], has dramatically transformed Chinese youth’s outlook for future. As uncertainties and outlooks turn bleak, many young college graduates are unable to find stable and well-paid jobs; they have to lower their job expectations and start taking up job positions that are not commensurate with their college education and credentials. College graduates have been increasingly reported to work in low-skilled, non-white-collar jobs such as food delivery, being a waiter or waitress, domestic work, or working in the assembly line. Diminishing job prospects triggered a brand-new job called “full-time children” who received a paycheck from their parents each month by running errands and caring for young siblings or older family members in exchange for moving back in and living with their family. Given China’s cultural values of respect and social harmony, it is possible that this tentative parent–child “employment relationship” lessened the strenuous labor market and contributed to the well-being of the young generation.

Using two types of analyses in sequential order, this study contributes to the literature by testing the psychometric properties of the well-being measure and its association with fairness and/or trust in society based on a nationally representative sample of participants. First, we used EFA to obtain a better understanding of the well-being measure. It is identified that the well-being measure has five independent dimensions: physical and mental health challenges, positive outlook, sense of purpose, personal growth, and self-fulfillment. By disentangling complex relationships among all the items in this instrument, factor analysis uncovers latent variables and makes well-being a psychometrically sound measurement. Furthermore, we conducted regression analyses to examine whether well-being is related to fairness and/or trust in society. Research findings identify that well-being relates to fairness and/or trust, especially those dimensions such as physical and mental health challenges and positive outlook. Young adults with poor physical and mental health were less likely to perceive society as fair or people as trustworthy. In contrast, a positive outlook is strongly associated with a high level of fairness and trust in society. This empirical evidence can generate profound implications for policy and practice. By identifying the multi-faceted nature of well-being measures, policy-makers, practitioners, and other stakeholders can call to action and implement programs and activities to improve youth well-being. Young people face unique challenges in their everyday lives, and they need programs that can assist them in overcoming obstacles and fostering constructive dialogues in schools, with their families, and in work environments to enhance their overall well-being and allow them to thrive in various aspects of their lives.

Future research on the happiness and well-being of young adults may expand to family dynamics such as parent–child relationships, marital relationships, and childbearing. Living in a traditional Confucian culture, young adults are inevitably exposed to their parents’ expectations of obtaining a degree or becoming a skilled worker, getting married, and having children. Because most Chinese parents pay their children’s educational costs, such as college tuition, books and supplies, and food and board, it is natural that children bond their parents with obedience. Family matters in terms of happiness and well-being. The intricate web of relationships, emotions, interactions, and engagement within a family unit can vary greatly depending on specific family members. Young adults’ abilities to deal with their parents, in-laws, spouses, and children, if possible, are likely to assume a vital role in shaping their level of happiness. It is especially common in China that parents continue to support their adult children financially. The mix of economic and non-economic family characteristics further causes challenging times among family members and becomes a source of differences in happiness. Additionally, once the new wave of CGSS data is released, we will navigate new variables and identify changes in well-being over time. By comparing results from two points of time (T1 vs. T2), we will be able to recognize the depth of well-being measurement.

There are some limitations to this study. First, the application of this study is limited in the context of China during the COVID-19 lockdown when zero-COVID policies were implemented across the nation and mass lockdowns were observed. Therefore, caution needs to be taken when generalizing research findings from this study to contemporary society. The pandemic has hit China hard economically, medically, and socially. After four decades of rapid economic development, China’s economic activities have slowed down to an annual growth rate of 2.2 percent in GDP in 2020, much lower than 6% in 2019 [52]. As China is still experiencing economic recovery and reopening, it is unclear how the social dimension of this structural transformation will change over time. Second, the cross-sectional nature of this study makes it impossible to establish causality between predictors and outcome variables. The snapshot only allows researchers to capture the pattern and association at one point in time.

## 6. Conclusions

Using the nationally representative data from the 2021 *Chinese General Social Survey* (CGSS), this study analyzed the happiness and well-being of 1726 young adults ages 18 to 34 years old living in 19 provinces in China. The research findings generated five factors from the well-being instrument and revealed the multi-faceted nature of subjective well-being. Those five latent constructs of happiness included physical and mental health challenges, a positive outlook, a sense of purpose, personal growth, and self-fulfillment. Physical and mental health have become the cornerstone for happiness daily. As young adults in this age range are experiencing many adaptive challenges such as transition to college life, graduation from college, work toward economic self-sufficiency, formation of an intimate relationship, and establishment of a new family, they must go through many stages of stressful moments. It is essential that they prepare themselves with existing and emerging resilience capabilities. In addition, this study identifies that happiness and well-being are associated with fairness and trust in society. On the one hand, young people with poor physical and mental health conditions are less likely to perceive that society is fair or that people are trustworthy. On the other hand, a positive outlook and future orientation are strongly associated with high fairness and/or trust. As China continues to expand its infrastructure on education, health, and other technology applications, both public and private sectors are engines of economic growth that can provide young people with more opportunities for upward mobility. A strong connection between happiness and well-being, fairness, and trust at a societal level is crucial for society’s long-term sustainability and prosperity.

## Figures and Tables

**Table 1:** Descriptive characteristics of youth (data: CGSS2021).

Variables	Frequency	Percentage	Mean (SD)
Age			26.39 (5.092)
Gender			
Male	778	45.1%	
Female	948	54.9%	
Ethnicity			
Han	1582	91.7%	
Minority	144	8.3%	
Education			
Middle school or less	365	21.2%	
High school or equivalent	407	23.6%	
Associate degree	286	16.6%	
College +	666	38.6%	
Self-reported health			
Poor or fair	347	20.1%	
Moderately healthy	781	45.3%	
Very healthy	596	34.6%	
Marital Status			
Never married	959	55.6%	
Other	767	44.4%	

**Table 2:** Descriptive statistics for the happiness instrument (data: CGSS2021).

Items	*n*	Min	Max	Mean (SD)
1, Society provides more opportunities	596	1	6	1.65 (0.903)
2, I have become more capable over the years	599	1	6	4.86 (0.730)
3, My life goals are fulfilling	597	1	6	4.74 (0.717)
4, I am mostly idle	597	1	6	2.89 (1.215)
5, I am unclear of how meaningful what I have done in my life is	595	1	6	2.75 (1.183)
6, I fear body functions	598	1	6	2.65 (1.196)
7, I am content compared to my peers	595	1	6	4.36 (0.997)
8, I am satisfied with my family income	598	1	6	3.92 (1.211)
9, I am worried about small things	601	1	6	3.13 (1.275)
10, I am upset with my physical health	601	1	6	2.56 (1.165)
11, I have a hard time maintaining friendship with others	600	1	6	2.48 (1.084)
12, I like my personality	597	1	6	4.28 (1.120)
13, I have fewer friends than most people.	595	1	6	2.95 (1.266)
14, I am happy staying with my family	600	1	6	4.80 (0.883)
15, I have worse luck than most people	593	1	6	2.74 (1.172)
16, I am confident with societal development	596	1	6	4.79 (0.826)
17, I am at a disadvantage compared to other	593	1	6	2.56 (1.020)
18, I lose spirit when encountering unhappy things	599	1	6	2.84 (1.221)
19, I have become more mature over time	598	1	6	4.73 (0.826)
20, I have difficult times with my family	597	1	6	3.09 (1.308)
21, I am satisfied with the natural environment	599	1	6	4.39 (1.047)

**Table 3:** Factor loadings for subjective happiness dimensions from factor analysis (data: CGSS2021).

Factors	Items	Original Statement	Factor1	Factor2	Factor3	Factor4	Factor5
Physical and mental challenges	6	I fear my body functions.	0.645				
	9	I am worried about small things.	0.576				
	10	I am upset with my physical health.	0.793				
	11	I have a hard time maintaining friendships with others.	0.684				
	13	I have fewer friends than most people.	0.551				
	15	I have worse luck than most people.	0.481				
	17	I am at a disadvantage compared to others.	0.582				
	18	I lose spirit when encountering unhappy things.	0.560				
Positive outlook	1	Society provides more opportunities.		0.621			
	2	I have become more capable over the years.		0.797			
	3	My life goals are fulfilling.		0.764			
	16	I am confident with societal development.		0.543			
Sense of purpose	4	I am mostly idle.			0.811		
	5	I am unclear of how meaningful what I have done in my life is.			0.753		
	20	I have difficult times with my family.			0.458		
Personal growth	12	I like my personality				0.680	
	14	I am happy staying with my family.				0.545	
	19	I have become more mature over time.				0.575	
	21	I am satisfied with the natural environment.				0.498	
Self-fulfillment	7	I am content compared to my peers.					0.612
	8	I am satisfied with my family income.					0.812

**Table 4:** Total variance explained from factor analysis (data: CGSS2021).

Factors	Factor Names	Eigenvalues	Percentage of Variance	Cumulative Percentage of Variance
1	Physical and mental challenges	5.268	25.085%	25.085%
2	Positive outlook	2.569	12.234%	37.319%
3	Sense of purpose	1.289	6.139%	43.458%
4	Personal growth	1.074	5.116%	48.574%
5	Self-fulfillment	1.023	4.870%	53.444%
Other	n/a	n/a	n/a	n/a

**Table 5:** Multiple regression examining the relationship between fairness and wellbeing.

Variables	Unstandardized Coefficient (Std. Err.)	Standardized Coefficients (Beta)	*p*-Value	95% CI (Lower Bound, Upper Bound)
Physical and mental challenges	−0.083(0.031)	−0.114	0.007	(−0.144, −0.023)
Positive outlook	0.106(0.030)	0.146	<0.001	(0.047, 0.166)
Sense of purpose	−0.045(0.031)	−0.063	0.137	(−0.105, 0.014)
Personal growth	0.046(0.030)	0.063	0.132	(−0.014, 0.106)
Self−fulfillment	0.088(0.031)	0.121	0.004	(0.028, 0.148)
Characteristics:				
Age	−0.001(0.009)	−0.005	0.934	(−0.018, 0.017)
Gender (1 = male, 0 = female)	−0.003(0.063)	−0.002	0.962	(−0.128, 0.121)
Education				
High school	−0.007(0.093)	−0.004	0.938	(−0.190, 0.176)
Associate	−0.014(0.104)	−0.007	0.893	(−0.219, 0.191)
College+	0.005(0.085)	0.004	0.950	(−0.161, 0.172)
Racial/Ethnicity (1 = Han, 0 = other)	0.035(0.108)	0.014	0.743	(−0.176, 0.247)
Health	0.062(0.042)	0.065	0.140	(−0.021, 0.145)
Marital status (1 = never married, 0 = other)	−0.001(0.090)	−0.001	0.993	(−0.177, 0.175)
Constant	2.329 (0.304)		<0.001	(1.733, 2.926)

**Table 6:** Multiple regression examining the relationship between trust and wellbeing.

Variables	Unstandardized Coefficient (Std. Err.)	Standardized Coefficients (Beta)	*p*-Value	95% CI (Lower Bound, Upper Bound)
Physical and mental challenges	−0.109(0.034)	−0.136	0.002	(−0.177, −0.042)
Positive outlook	0.093(0.034)	0.115	0.006	(0.027, 0.159)
Sense of purpose	−0.084(0.034)	−0.104	0.014	(−0.150, −0.017)
Personal growth	0.044(0.034)	0.055	0.190	(−0.022, 0.111)
Self−fulfillment	0.004(0.034)	0.005	0.910	(−0.063, 0.071)
Characteristics:				
Age	−0.011(0.010)	−0.064	0.281	(−0.030, 0.009)
Gender (1 = male, 0 = female)	0.001(0.070)	0.001	0.988	(−0.137, 0.139)
Education				
High school	0.083(0.104)	0.043	0.422	(−0.120, 0.287)
Associate	0.104(0.116)	0.046	0.371	(−0.124, 0.332)
College+	0.195(0.095)	0.119	0.040	(0.009, 0.381)
Racial/Ethnicity (1 = Han, 0 = other)	0.121(0.120)	0.042	0.312	(−0.114, 0.356)
Health	−0.029 (0.047)	−0.027	0.541	(−0.121, 0.063)
Marital status (1 = never married, 0 = other)	−0.023 (0.100)	−0.014	0.819	(−0.218, 0.173)
Constant	2.586 (0.337)		<0.001	(1.923, 3.248)

## Data Availability

This study utilizes secondary data that are available for public use.
